# Optimizing photoperiod for growth and centellosides biosynthesis in *Centella asiatica* under vertical farming conditions

**DOI:** 10.1038/s41598-026-44883-w

**Published:** 2026-03-22

**Authors:** Gyu-Sik Yang, In-Je Kang, Han-Sol Sim, Seong-Nam Jang, Ga Oun Lee, Kyoung-Ok Choi, Jun Gu Lee, Yu Kyeong Shin, Thanh-Tam Ho, Ki-Ho Son

**Affiliations:** 1 Division of Horticultural Science, Gyeongsang National University, Jinju, 52725 Republic of Korea; 2 Department of GreenBio Science, Gyeongsang National University, Jinju, 52725 Republic of Korea; 3 Department of Horticulture, College of Agriculture & Life Sciences, Jeonbuk National University, Jeonju, 54896 Korea; 4 Institute for Global Health Innovations, Duy Tan University, Da Nang, 550000 Viet Nam; 5 Biotechnology Department, College of Medicine and Pharmacy, Duy Tan University, Da Nang, 550000 Viet Nam

**Keywords:** Antioxidants, Energy-use efficiency, Extended light period, Growth characteristics, Medicinal plant, Oxidative stress, Biochemistry, Biotechnology, Physiology, Plant sciences

## Abstract

This study aimed to identify the optimal photoperiodic conditions for *Centella asiatica*, a medicinal plant known for its high-value bioactive compounds, including madecassoside and asiaticoside, in a vertical farming system. *C. asiatica* plants were cultivated for 4 weeks under varying photoperiods [20/4, 16/8, 12/12, and 8/16 h (light/dark)] with a light intensity of 200 ± 10 µmol·m^− 2^·s^− 1^. The findings revealed that shoot fresh and dry weights, leaf length, leaf area, and leaf width were highest under 20/4, 16/8, and 12/12 h, while the number of runners peaked at 12/12 h. However, physiological disorders were observed under 20/4 and 16/8 h. Total phenol, total flavonoid, and antioxidant capacity increased linearly with photoperiod, reaching their maximum at 20/4 h and minimum at 8/16 h. Madecassoside and asiaticoside concentrations increased when the photoperiod exceeded 12/12 h, while madecassic acid and asiatic acid concentrations were higher under 8/16 h. Furthermore, light-use efficiency and energy-use efficiency, energy efficiency metrics, were highest at 12/12 h compared to other treatments. These findings indicate that the optimal photoperiod for maximizing biomass and secondary metabolite production in *C. asiatica* grown under 200 µmol·m^− 2^·s^− 1^ white light-emitting diodes in vertical farms is 12/12 h.

**Supplementary Information:**

The online version contains supplementary material available at 10.1038/s41598-026-44883-w.

## Introduction

As carbon emissions continue to rise, extreme weather events extreme weather events and global warming are projected to decrease crop yields and reduce cultivated areas, prompting an ongoing search for alternative crops to replace conventional ones^1^. In these constrained environments, closed vertical farms are emerging as a promising solution for flexible and reliable production of diverse crops^2^. Vertical farms rely on artificial lighting to simulate sunlight, and use either soil-based or hydroponic systems to grow crops year-round under fully controlled environmental conditions^3^. While these farms require relatively relatively high cultivation costs they are gaining recognition for their eco-friendly attributes and their capacity to produce fresh, high-value crops^4,5^. However, efficient resource utilization in vertical farms remains critical, underscoring the need to optimize energy consumption while managing complex environmental variables^6^. Therefore, numerous studies have investigated the relationships between light-use efficiency (LUE) and energy-use efficiency (EUE) with plant fresh and dry weights in vertical farming systems^7,8^.

Artificial lighting is a key environmental factor in vertical farming, and optimizing its parameters—including illuminance, spectrum, and photoperiod—is essential for maximizing both crop productivity and energy efficiency. Light regulation, encompassing photoperiod, intensity, and quality, plays a critical role in plant growth and metabolite biosynthesis^9^. To maximize crop yields, it is crucial to optimize light intensity and photoperiod by balancing production time and lighting costs^10^. Research on light intensity has revealed that excessive levels, such as 800 µmol·m^− 2^·s^− 1^ for lettuce, can lead to reduced chlorophyll fluorescence indicators—Fv/Fm, qP, ΦPSII, and electron transport rate (ETR)–and lower yields. Accordingly, a light intensity of 400–600 µmol·m⁻²·s⁻¹ has been recommended as the optimal range^11^. Studies on photoperiods have indicated that longer photoperiods in lettuce can lead to higher carbohydrate accumulation, accelerating growth rates and enhancing pigmentation, thereby improving crop quality^12,13^. However, several studies have suggested that evaluating light factors in combination rather than alone is crucial for optimizing plant growth. Reducing photosynthetic photon flux density (PPFD) and extending the photoperiod under the same daily light integral (DLI) can improve photosystem II efficiency and increased fresh and dry weight in lettuce^14^. This combination of longer photoperiods and lower light intensity also resulted in higher fresh and dry weights in lettuce. However, the relationship between photoperiod in the medicinal plant *Centella asiatica* is still unclear.

*Centella asiatica* has been recognized as a medicinal panacea plant for over 3,000 years^15^. Belonging to the Apiaceae family, it is a perennial herb native to tropical and subtropical regions worldwide. Its medicinal value lies in its primary bioactive compounds, such as madecassoside, asiaticoside, madecassic acid, and asiatic acid, which exhibit properties effective in wound healing, anti-aging, antibacterial, anticancer, and venous disease treatments^16,17^. Additionally, due to its role in collagen synthesis, *C. asiatica* extracts are highly effective for skin applications, garnering significant interest in the cosmetics industry^18^. However, wild *C. asiatica* often shows low purity and inferior quality in its bioactive components^19^. Moreover, the increasing demand for *C. asiatica* in cosmetics and therapeutics necessitates the development of sustainable and economically efficient production strategies to meet market requirements^20,21^.

Several studies have aimed to address the growing demand for *C. asiatica* extracts while optimizing cultivation time and energy use. Research has shown that high photosynthetically active radiation (PAR) intensity induces protective mechanisms, such as oxidative stress, in *C. asiatica*^22^. Song et al. reported that the red-to-blue light ratio in LED systems significantly affects *C. asiatica* growth and centellosides content, with 200 µmol·m^− 2^·s^− 1^ identified as optimal for its growth^23^. In addition to terpenoids such as centellosides, *C. asiatica* contains phenolic compounds and flavonoids, which exhibit strong antioxidant, anticancer, and antimicrobial activities. These metabolites also serve important protective functions and should be considered alongside terpenoids when assessing the plant’s metabolic responses to environmental factors. Another study demonstrated that higher light intensity promotes *C. asiatica* growth and centellosides production, while lower light intensities negatively affect grwoth^24^. Additionally, Shawon et al. found that the highest growth and centellosides content in *C. asiatica* were observed when a nutrient solution with a concentration of 1.2 dS·m^− 1^ was used^25^. Despite these insights, no foundational research has been conducted on the effects of different photoperiods on *C. asiatica*.

In *C. asiatica*, terpenoids such as centellosides are the primary bioactive compounds with medicinal importance, while phenolic compounds and flavonoids also play valuable defensive roles that enhance the plant’s functional quality. Moreover, variations in photoperiod alter the DLI, thereby influencing carbon fixation and the synthesis of these metabolites. Therefore, elucidating this relationship is essential for improving both biomass productivity and metabolite quality. This study thus aimed to investigate the effects of different photoperiods on the growth and bioactive compound accumulation of *C. asiatica* under controlled conditions and to identify the optimal photoperiod and DLI for efficient, high-quality production.

## Materials and methods

### Plant material and growth conditions

**Fig. 1:**
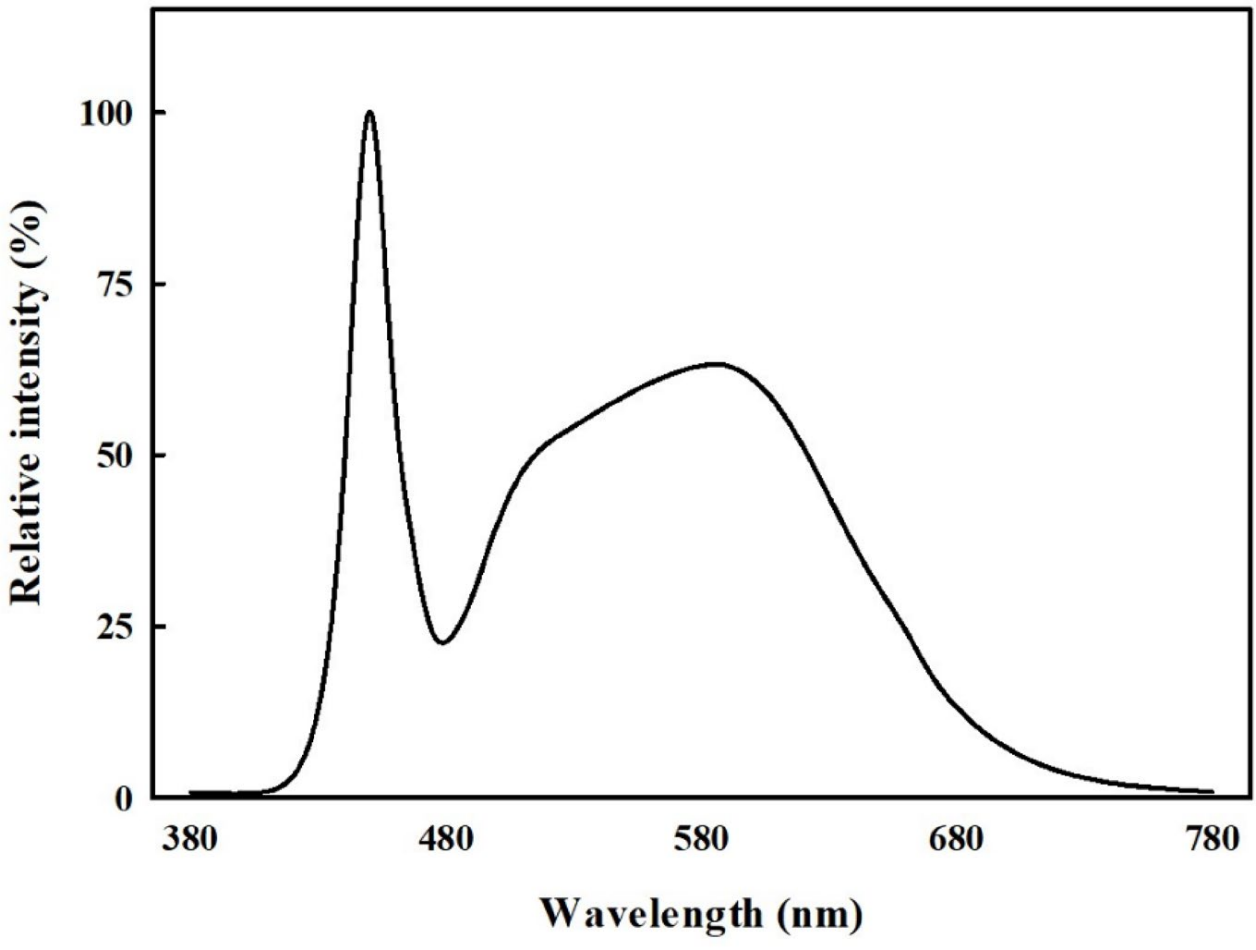
The relative intensity of the light-emitting diode (LED) light sources used in this study.

The plant material used in this study was *C. asiatica* cultivated at the Nae Dong Campus of Gyeongsang National University (Gyeongsangnam-do, Korea; 35°09’38"N, 128°04’39"E; altitude 44 m). Uniform plants with three leaves were selected and acclimatized in distilled water for one week. Acclimatization was conducted using sponges (ø 60 mm × height 30 mm) and hydroponic pots (ø 65 mm × height 75 mm). The objective of this study was to evaluate crop responses under realistic lighting conditions that are more economical and operationally feasible for large-scale production. Accordingly, white LEDs were used to reflect lighting conditions commonly used in commercial or farm-scale vertical farming systems, and the acclimation environment was maintained at 100 photosynthetic photon flux density (PPFD) using cool white LEDs (JS-HLT30; Zhong Shan Jinsung Electronic Co., Zhongshan, China) (Fig. 1). Temperature and relative humidity were set at 24.0 °C ± 0.7 °C and 60% ± 5%, respectively. For the experiment, two plants were grouped as one unit, with 10 groups per treatment. The plants were transplanted into a circulating hydroponic system utilizing the deep flow technique. The hydroponic system was designed as a four-tier structure to simulate vertical farming conditions, with each photoperiod treatment placed in a different layer to minimize light interference. The shortest photoperiod treatment was placed on the top layer, and the remaining treatments were placed sequentially in the layers below it (see 2.2 for details). Environmental conditions, including temperature (24 °C ± 0.7 °C) and relative humidity (60% ± 5%), were kept constant. Hoagland’s solution (40 L; pH 6.0 ± 0.1, EC 1.0 ± 0.1 dS·m^− 1^) was supplied immediately after transplanting and refreshed every 14 days.

### Light treatment

White LEDs (JS-HLT30; Zhong Shan Jinsung Electronic Co., Zhongshan, China) (Fig. 1) provided light for a period of 4 weeks, with photoperiods set at a uniform light intensity of 200 µmol·m^− 2^·s^− 1^. The treatments included four photoperiod regimes: (1) 20 h light/4 h dark (20/4 h), (2) 16 h light/8 h dark (16/8 h), (3) 12 h light/12 h dark (12/12 h), and (4) 8 h light/16 h dark (8/16 h) (Table 1). Light intensity was measured using a light spectrum analyzer (LI-180 Spectrometer; LI-COR, Lincoln, NE, USA). Growth pots were rearranged systematically every 2 days to ensure uniform light distribution across treatments.

**Table 1:** Light conditions (20 h light/4 h dark; 20/4 h, 16 h light/8 h dark; 16/8 h, 12 h light/12 h dark; 12/12 h, and 8 h light/16 h dark; 8/16 h) used to evaluate the effect of various photoperiods on the growth and development of *Centella asiatica*.

Photoperiod (day/night)	Light intensity (µmol·m^− 2^·s^− 1^)	Daily light integral (mol·m^− 2^·d^− 1^)
20/4 h	200	14.40
16/8 h	200	11.52
12/12 h	200	8.64
8/16 h	200	5.76

### Growth measurements

After 4 weeks, growth measurements were conducted using nine plants randomly selected from each treatment group. Shoot (including stolons) and root fresh weights were measured for each plant (g plant^− 1^) using an electronic microbalance (PX224KR/E; OHAUS, NJ, USA). Dry weight was determined by drying samples in an oven (WOF-155; DAIHAN, Gangwon, Korea) at 70 °C for 3 days. Leaf length and width were measured on the three largest leaves using a vernier caliper (CD-P30S; Mitutoyo Co., Kawasaki, Japan), and the mean values were recorded. Leaf area was assessed using a LI-3100 C leaf area meter (LI-COR, Lincoln, NE, USA). The number of leaves and runners was also recorded. The specific leaf area (SLA) of each plant was calculated using the following formula:

$$\:\mathrm{S}\mathrm{L}\mathrm{A}\:=\frac{\mathrm{L}\mathrm{e}\mathrm{a}\mathrm{f}\:\mathrm{a}\mathrm{r}\mathrm{e}\mathrm{a}\:\left({\mathrm{c}\mathrm{m}}^{2}\right)}{\mathrm{L}\mathrm{e}\mathrm{a}\mathrm{f}\:\mathrm{d}\mathrm{r}\mathrm{y}\:\mathrm{w}\mathrm{e}\mathrm{i}\mathrm{g}\mathrm{h}\mathrm{t}\:\left(\mathrm{g}\right)}$$

### Bioactive compounds

To analyze the total phenol (TP) concentration in *C. asiatica* under different light treatments, powdered leaf samples collected 4 weeks after treatment initiation were used. The analysis was conducted using a modified method based on a previously reported protocol^26^. Approximately 0.015 g of powdered sample was extracted with 1.5 mL of 80% (v/v) acetone, sonicated for 15 min, and stored at 4 °C for 12 h. The extract was then centrifuged at 1,350 rpm for 2 min at 25 °C, and the supernatant was collected. For the assay, 135 µL of distilled water, 750 µL of 10% diluted Folin–Ciocalteau reagent, 50 µL of the sample extract (or 50 µL of 80% acetone as a control), and 600 µL of 7.5% Na_2_CO_3_ were sequentially added to a microtube and vortexed for 10 s. The samples were incubated in a 45 °C water bath for 15 min and then cooled. The absorbance was measured at 765 nm using a spectrophotometer (Libra S32, Biochrom Ltd., Cambridge, UK). A standard curve was prepared using gallic acid, and the total phenol concentration was expressed as milligrams of gallic acid equivalent per gram of dry weight (mg GAE·g^− 1^ DW).

The total flavonoid (TF) concentration was determined following a modified method based on a previously reported protocol^26^. For the extraction, approximately 0.05 g of powdered dried samples of leaves from the four-week light treatments was mixed with 4 mL of 70% (v/v) ethanol. The mixture was briefly vortexed, sonicated for 15 min, and centrifuged at 1,350 rpm for 2 min at 25 °C to obtain the supernatant. The assay mixture was prepared by sequentially adding 1.25 mL of distilled water, 75 µL of 5% sodium nitrite (NaNO_2_), and 250 µL of the extract (or 250 µL of 70% ethanol as a blank) and allowing it to react for 5 min. Subsequently, 150 µL of 10% aluminum chloride (AlCl_3_) was added and incubated for 6 min. Next, 500 µL of 1 M sodium hydroxide (NaOH) and 275 µL of distilled water were added, and the mixture was vortexed briefly. The absorbance was measured at 510 nm using a spectrophotometer (Libra S32, Biochrom Ltd., Cambridge, UK) after 5 min. The total flavonoid concentration was expressed as milligrams of catechin hydrate equivalents per gram of dry weight (mg CHE·g^− 1^ DW).

The antioxidant capacity of *C. asiatica* dried leaves was assessed using a non-enzymatic 2,2′-azino-bis(3-ethylbenzothiazoline-6-sulfonic acid) (ABTS) radical scavenging assay, modified from a previously reported protocol^26^. Enzymatic antioxidants such as superoxide dismutase (SOD) or catalase (CAT) were not included in the analysis. To prepare the extracts, 0.015 g of leaf powder was mixed with 1.5 mL of 80% (v/v) acetone, sonicated for 15 min, and stored at − 20 °C in the dark. The mixture was centrifuged at 1,000 rpm for 2 min at 25 °C, and the supernatant was collected for analysis. An ABTS stock solution was prepared by dissolving 13.7 mg of ABTS in 10 mL of distilled water to achieve a 2.5 mM concentration. To activate the ABTS, 0.4 g of manganese dioxide (MnO_2_) was added, and the mixture was stirred for 30 min. The solution was sequentially filtered through standard filter paper and a 0.2-µm syringe filter. The filtered ABTS solution was incubated in a 30 °C water bath in a conical tube. Absorbance was measured at 730 nm using a spectrophotometer (Libra S32, Biochrom Ltd., Cambridge, UK). The ABTS solution was diluted with 5 mM phosphate-buffered saline to adjust its absorbance to 0.7 ± 0.05. For the assay, 100 µL of the sample extract was mixed with 1 mL of the adjusted ABTS solution, vortexed briefly, and allowed to react for 1 min before measuring the absorbance. Antioxidant activity was expressed as millimoles of Trolox equivalent antioxidant capacity (TEAC) per gram of dry weight (mg TEAC·g^− 1^ DW).

### Centellosides concentration

In a modified approach based on a previously reported protocol^23^, centellosides concentrations—including madecassoside, asiaticoside, madecassic acid, and asiatic acid—were analyzed using high-performance liquid chromatography (HPLC) with an Agilent 1260 system (Agilent Technologies Inc., California, USA). A 0.05 g dry powdered sample of leaves was extracted using 3 mL of 80% methanol (MeOH) for 1 h, followed by centrifugation to collect the supernatant, which was filtered through a 0.45 μm PVDF filter. Analysis employed a Nova-Pak C18 column (3.9 × 150 mm, 4 μm, Waters Corp., Milford, MA, USA), with 100% acetonitrile (solvent A) and 100% distilled water (solvent B). The centellosides concentration was measured at 210 nm using a UV detector under a 20%−100% linear gradient at a flow rate of 1.0 mL/min at 30 °C for 25 min. All experiments were conducted in triplicate, and results were expressed as mean values.

**Fig. 2:**
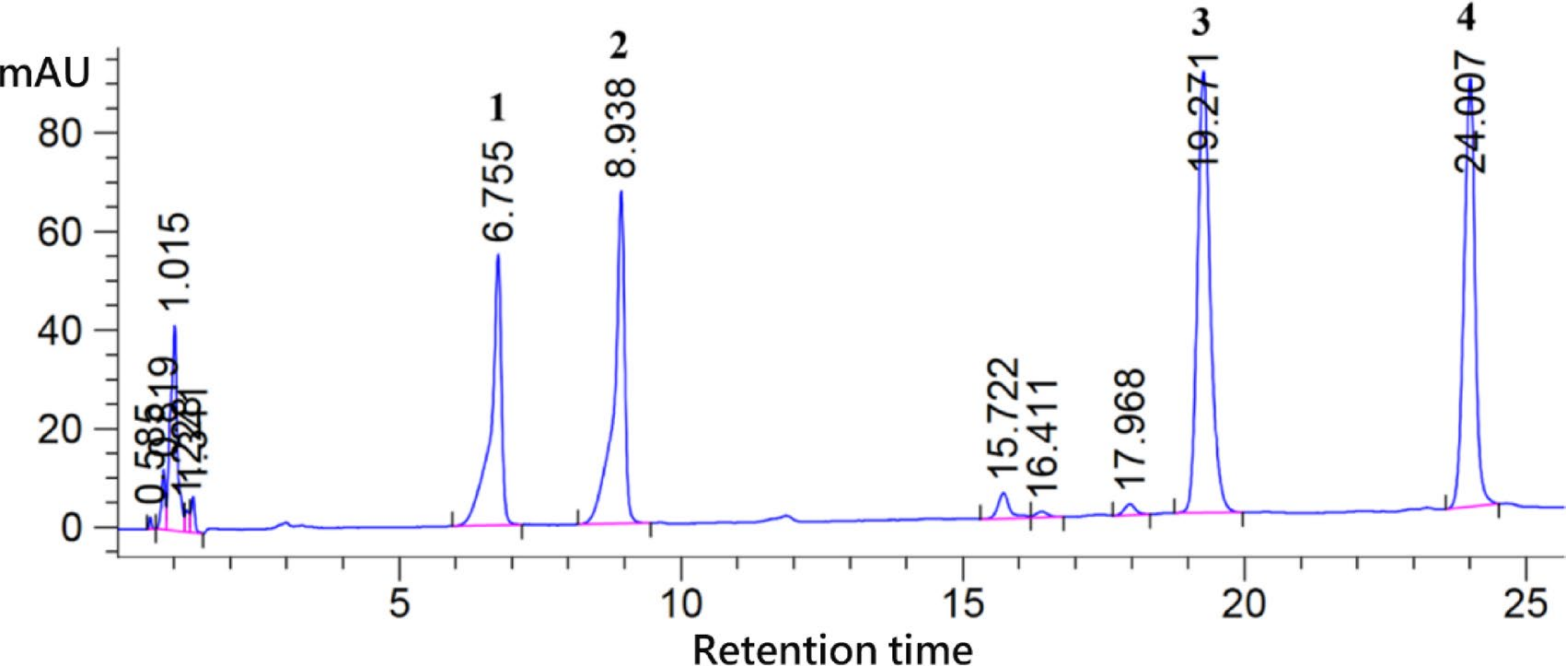
Centellosides compound in the standard mixture analyzed using high-performance liquid chromatography. Peaks in the chromatogram represent the following compounds: (1) madecassoside; (2) asiaticoside; (3) madecassic acid; and (4) asiatic acid.

Calibration curves for each compound were constructed using five standard concentrations. The regression equations were derived from the standard curves shown in Fig. 2, and were as follows:

Madecassoside: B = 4.445 A + 26.54 (R² = 0.989).

Asiaticoside: B = 3.828 A + 45.11 (R² = 0.995).

Madecassic acid: B = 6.417 A + 108.52 (R² = 0.997).

Asiatic acid: B = 6.634 A + 60.44 (R² = 0.998).

where B is the compound concentration (mg·g⁻¹ DW), and A is the peak area obtained from the HPLC chromatogram. The chemical structures used in this study can be found in the supplementary materials (Fig. S1).

### Light and EUE

Light use efficiency (LUE) was calculated using a modified version of the method described by Son et al.^27^, measuring LUE as the total dry weight per measured PPFD. Similarly, Energy use efficiency (EUE) was calculated using an adapted approach based on Pennisi et al.^28^, where EUE was calculated as power per hour per measured dry weight using the following formula:

$$\:\mathrm{L}\mathrm{U}\mathrm{E}\:=\frac{DW}{PPFD}$$

where DW is the average dry weight of the samples (g); PPFD is the photosynthetic photon flux density (µmol·m^− 2^·s^− 1^).

$$\:\mathrm{E}\mathrm{U}\mathrm{E}\:=\frac{DW}{h\cdot\:W}$$

where DW is the average dry weight of the samples (g), h is the h (h), and the electric energy consumed by the LED (kW).

### Statistical analysis

For growth traits, nine plants per treatment across biological replications were assessed for shoot and root fresh/dry weights, number of leaves and runners, leaf area, leaf length, and leaf width. Additionally, three plants per treatment across technical replications were used to evaluate TP, TF, ABTS, and centellosides concentration. Mean separation was conducted using Duncan’s multiple range test (*: Significant at *p* < 0.05, **: Significant at *p* < 0.01, ***: Significant at *p* < 0.001), and statistical analyses were performed using SAS software (version 9.45, Cary, NC, USA, SAS Institute Inc.). All figures and tables report means ± standard deviations. To analyze relationships among variables in *C. asiatica* plants, principal component analysis (PCA) was conducted using the FactoMine package in R software (version 4.1.1). PCA results highlighted the contributions of ADP, BDP, IER, and RF to each variable. Statistical assumptions for ANOVA were considered during analysis, and the method was deemed appropriate based on the experimental design and data distribution.

## Results

### Growth characteristics

**Fig. 3:**
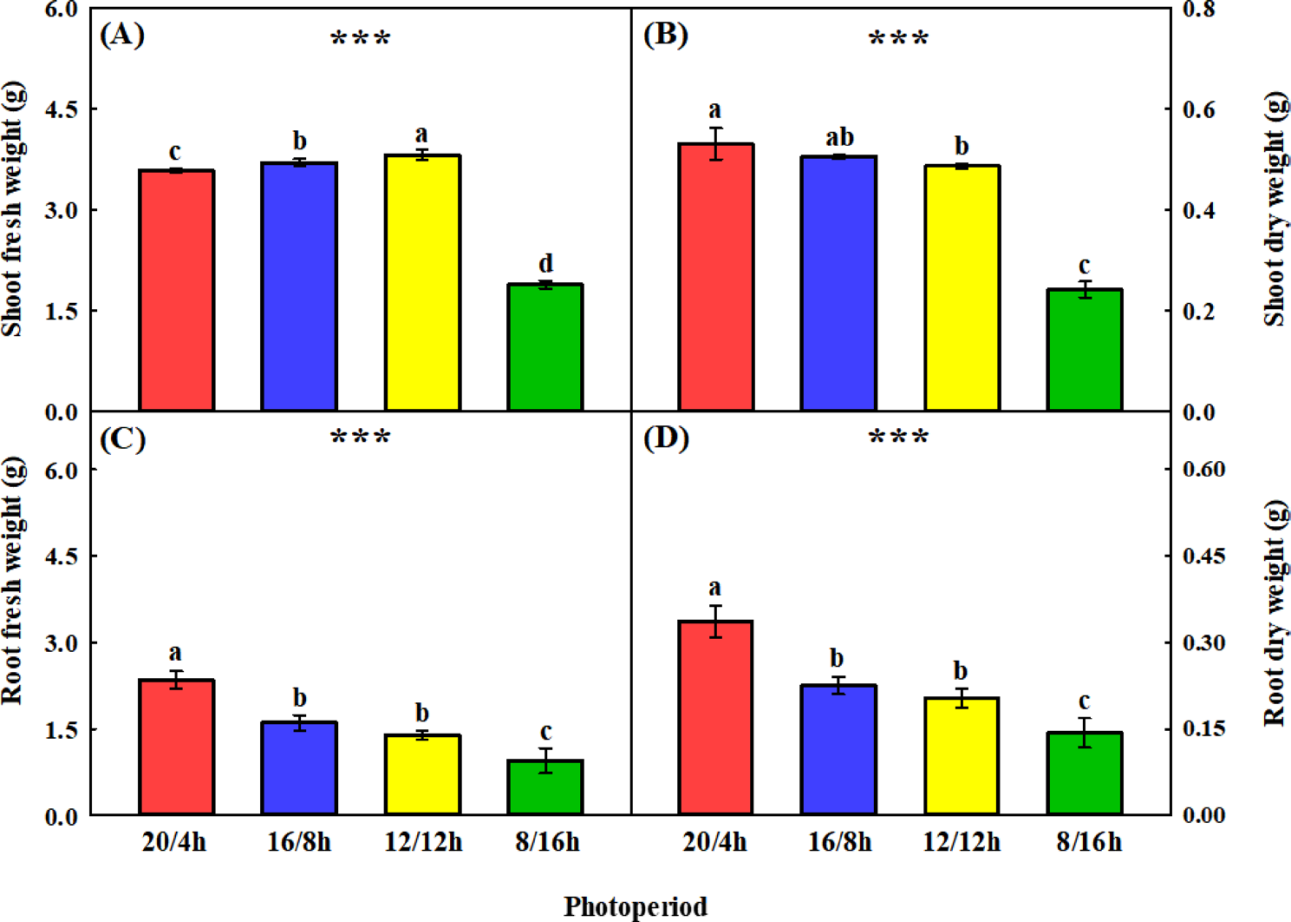
Shoot and root fresh and dry weights of *Centella asiatica* grown under different photoperiods (20 h light/4 h dark; 20/4 h, 16 h light/8 h dark; 16/8 h, 12 h light/12 h dark; 12/12 h, and 8 h light/16 h dark; 8/16 h) for 4 weeks. Panels **(A)–(D)** represent fresh and dry weights. Different letters above the bars indicate significant differences among the means (*p* < 0.05). Error bars represent the standard deviation (*n* = 9).

**Fig. 4:**
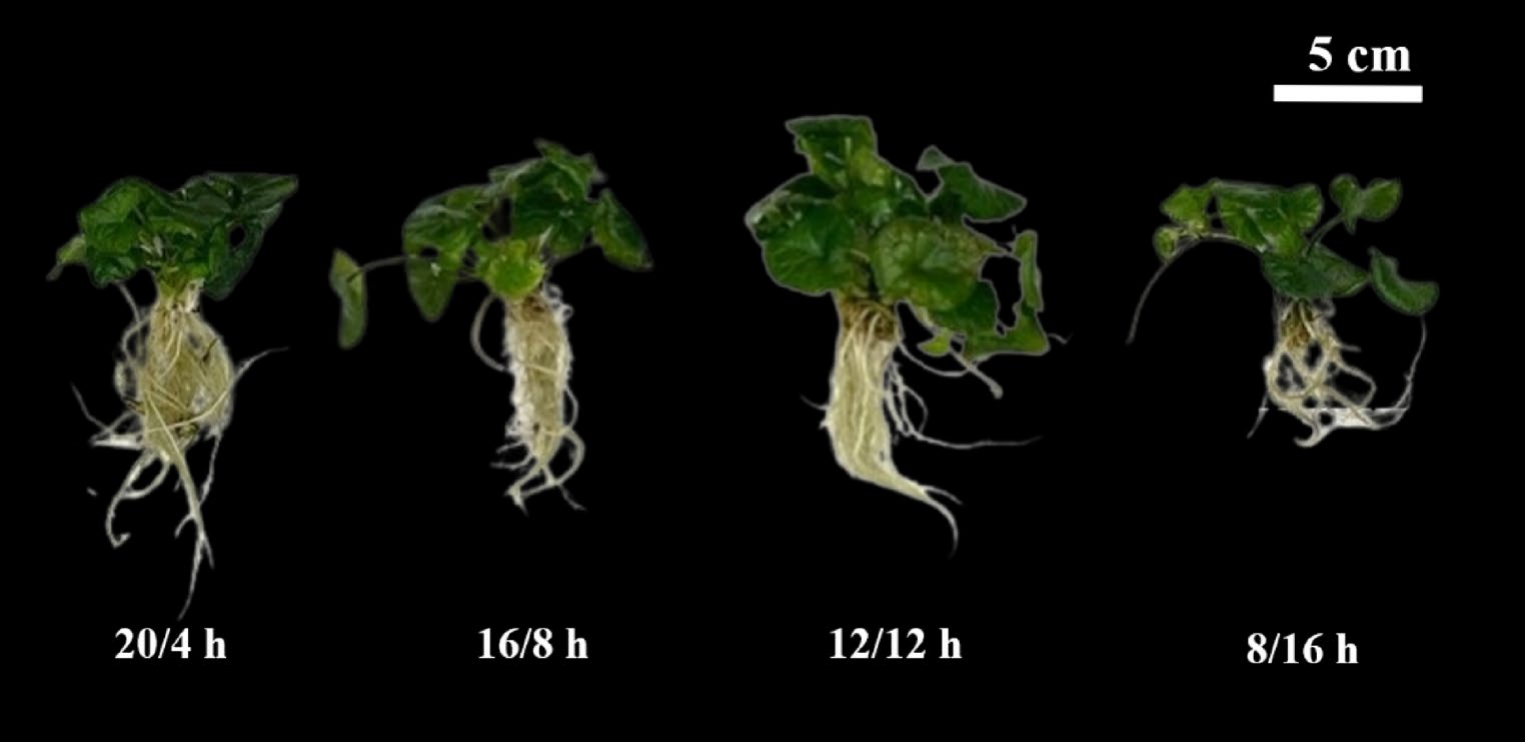
Picture of *Centella asiatica* under various photoperiods (20 h day/4 h night, 16 h day/8 h night, 12 h day/12 h night, and 8 h day/16 h night) after 4 weeks of cultivation.

Photoperiod variations significantly influenced the shoot and root growth of *C*. *asiatica* (Figs. 3 and 4). Growth parameters generally increased with extended photoperiods, while the lowest values observed under the 8-h photoperiod. Shoot fresh weight showed a marked increase at photoperiods of 12 h and longer, reaching its peak at 12/12 h, representing increases of 6.05%, 2.86%, and 50.54% compared to 20/4, 16/8, and 8/16 h, respectively. In contrast, stress symptoms such as leaf darkening and physiological disorders were observed in the 20/4 and 16/8 h treatments, with the most severe symptoms appearing under the 20/4 h photoperiod (Fig. S2). Root fresh and dry weights followed a different pattern, peaking at the 20/4 h photoperiod and reaching their lowest values under the 8/16 h photoperiod.

### Leaf characteristics

Leaf morphological traits of *C. asiatica* were influenced by photoperiod treatments (Table 2). Leaf length and area were significantly greater under the 12/12 h and 20/4 h photoperiods. Although leaf width showed no significant differences among treatments, an increase in leaf length contributed to overall leaf area. SLA, a key indicator of leaf thickness and photosynthetic investment, was highest under the 8/16 and 12/12 h, indicating thinner leaves and potentially higher light capture efficiency under moderate and shorter photoperiods. In contrast, SLA values were lower under the 16/8 and 20/4 h, suggesting thicker leaf structures.

**Table 2:** Effect of different photoperiods (20 h light/4 h dark; 20/4 h, 16 h light/8 h dark; 16/8 h, 12 h light/12 h dark; 12/12 h, and 8 h light/16 h dark; 8/16 h) on the growth and development of *Centella asiatica* (*n* = 9).

Photoperiod (day/night)	Leaf					Specificleaf area(cm^2^·g^− 1^)		Number of Leaves		Number of runners
	Length (cm)	Width (cm)		Area(cm^2^)						
20/4 h	2.10 ± 0.09 ^z^ a^y^	3.74 ± 0.19 a		69.43 ± 8.12 ab		6.90 ± 0.66 b		23.11 ± 4.35 a		0.56 ± 0.69 b
16/8 h	1.88 ± 0.08 b	3.38 ± 0.30 a		64.40 ± 2.50 b		6.95 ± 0.07 b		27.78 ± 2.22 a		1.56 ± 0.69 b
12/12 h	2.11 ± 0.07 a	3.72 ± 0.20 a		79.72 ± 6.90 a		8.17 ± 0.20 b		22.44 ± 3.60 a		2.67 ± 0.33 a
8/16 h	1.91 ± 0.18 ab	3.33 ± 0.17 a		45.58 ± 3.00 c		21.91 ± 10.31 a		14.11 ± 0.84 b		1.11 ± 0.38 b

^z^Means ± standard deviation (SD); ^y^Means with different letters within a column are significantly different (*p* < 0.05).

Leaf counts were higher under 12 h and longer photoperiods compared to the 8/16 h, with the highest number observed in the 12/12 h. However, longer photoperiods, such as 20/4 h, did not result in further increases. The number of runners was highest in 12/12 h, but no significant differences were observed between the different treatments.

### Bioactive compounds

This study found that TP, TF, and ABTS levels tended to increase with longer photoperiods (Table 3). In particular, the 20/4 h promoted the accumulation of antioxidant-related compounds in *C. asiatica*, indicating that photoperiod influences the biosynthesis of specialized metabolites. TP content was highest under the 20/4 h (21.17 ± 0.62 mg GAE·g⁻¹ DW), followed by the 16/8 and 12/12 h, and lowest under the 8/16 h treatment (13.66 ± 0.18 mg GAE·g⁻¹ DW). Similarly, TF content was significantly elevated under longer photoperiods, with the highest value recorded at 20/4 h (8.73 ± 0.07 mg CHE·g⁻¹ DW), and the lowest at 8/16 h (6.42 ± 0.19 mg CHE·g⁻¹ DW). ABTS radical scavenging activity followed a slightly different pattern. The highest antioxidant capacity (16.22 ± 0.06 mg TEAC·g⁻¹ DW) was observed under the 20/4 h treatment. However, no significant differences were observed among the 20/4 and 16/8 h and 8/16 h treatments.

**Table 3:** Effect of different photoperiods (20 h light/4 h dark; 20/4 h, 16 h light/8 h dark; 16/8 h, 12 h light/12 h dark; 12/12 h, and 8 h light/16 h dark; 8/16 h) on the total phenol (TP) and total flavonoid (TF) concentrations and the antioxidant activities (ABTS assay) of *Centella asiatica* at 4 weeks (*n* = 3).

Photoperiod (day/night)	TP (mg GAE·g^− 1^ DW)	TF (mg CHE·g^− 1^ DW)	ABTS (mg TEAC·g^− 1^ DW)
20/4 h	21.17 ± 0.62^z^ a^y^	8.73 ± 0.07 a	16.22 ± 0.06 a
16/8 h	20.38 ± 0.16 b	7.96 ± 0.19 b	16.08 ± 0.17 ab
12/12 h	19.26 ± 0.09 c	7.56 ± 0.08 c	15.78 ± 0.13 c
8/16 h	13.66 ± 0.18 d	6.42 ± 0.19 d	15.92 ± 0.06 bc
Significant	***	***	**

^z^Means ± standard deviation (SD); ^y^Means with different letters within a column are significantly different (*p* < 0.05). **: *p* < 0.01, ***: *p* < 0.001.

### Centellosides concentration

**Fig. 5:**
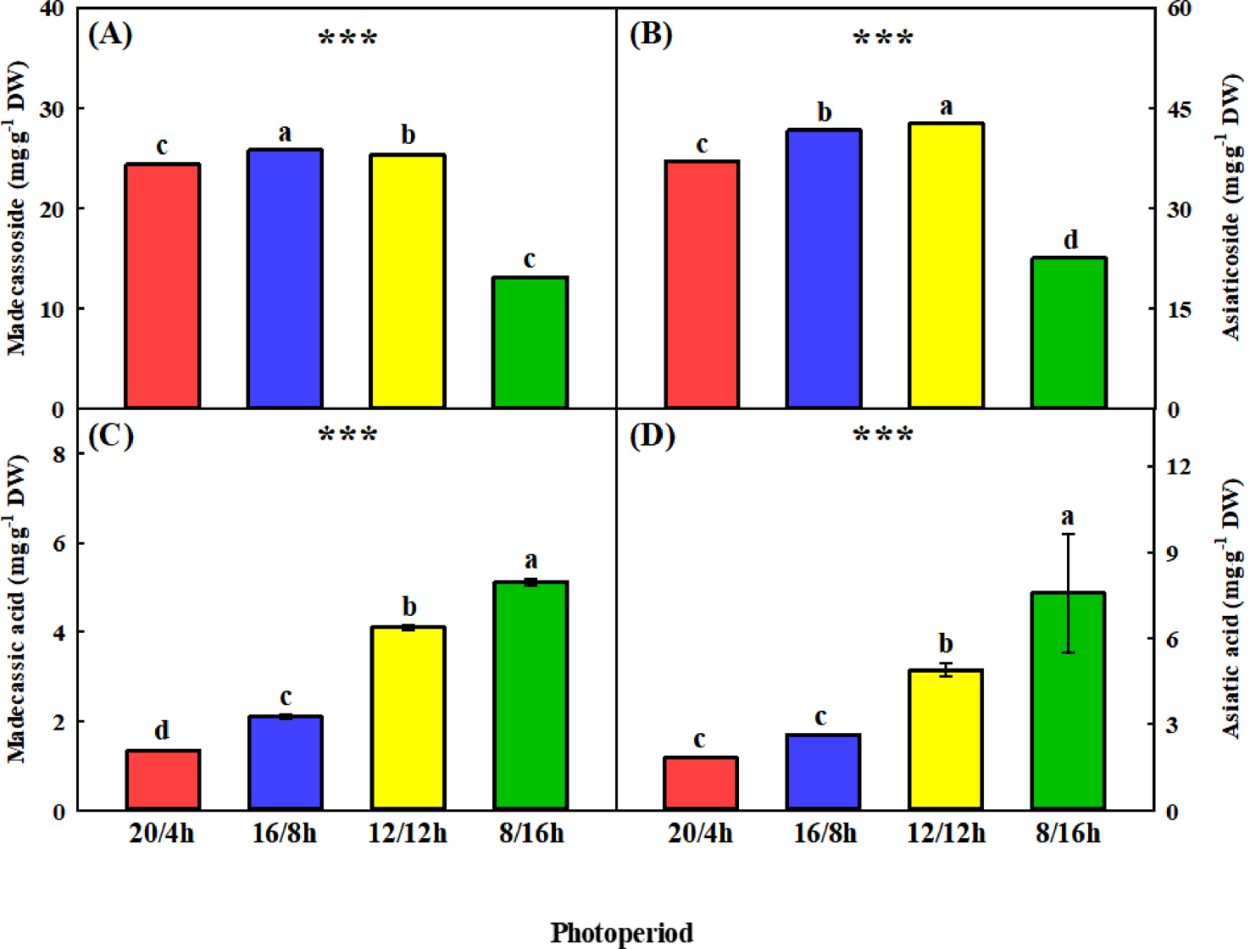
Centellosides concentration in *Centella asiatica* grown under various photoperiods (20 h light/4 h dark; 20/4 h, 16 h light/8 h dark; 16/8 h, 12 h light/12 h dark; 12/12 h, and 8 h light/16 h dark; 8/16 h) for 4 weeks. Panels **(A)–(D)** show the concentrations of madecassoside, asiaticoside, madecassic acid, and asiatic acid, respectively. Different letters above the bars indicate significant differences among the means (*p* < 0.05). Error bars represent the standard deviation (*n* = 3); in cases where they are not visible, the variation was too small to be discernible.

The concentrations of centellosides responded differently to changes in photoperiod (Fig. 5). Madecassoside peaked at 16/8 h, followed by 12/12 and 20/4 h, with the lowest levels at 8/16 h (Fig. 5A). For asiaticoside, the highest concentration was observed at 12/12 h, followed by 16/8 h and 20/4 h, with the lowest concentration at 8/16 h (Fig. 5B). In contrast, madecassic acid and asiatic acid were more abundant under shorter photoperiods, with the highest levels observed under 8/16 h (Fig. 5C, D). These results indicate that photoperiod influences centelloside biosynthesis differently depending on compound type with shorter days favoring acid accumulation and longer days tending to increase glycoside content.

### LUE and EUE

**Fig. 6:**
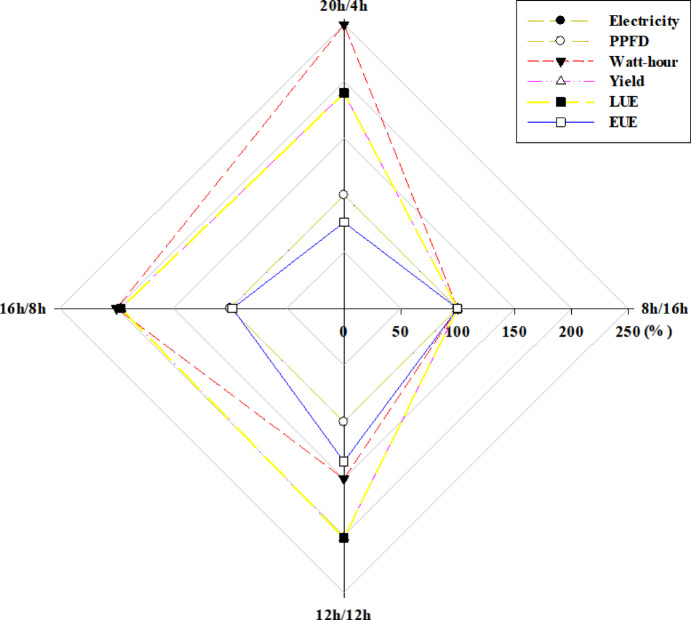
Power, photosynthetic photon flux density (PPFD), total power consumption, yield, light-use efficiency, and energy-use efficiency after 4 weeks of cultivation of *Centella asiatica* under different photoperiods (20 h light/4 h dark; 20/4 h, 16 h light/8 h dark; 16/8 h, 12 h light/12 h dark; 12/12 h, and 8 h light/16 h dark; 8/16 h).

Among photoperiod treatments, the 8/16 h was used as a relative baseline for calculating total consumed power, yield, LUE, and EUE under identical power and PPFD conditions (Fig. 6, Table S2). Total consumed power was 1.5 to 2.5 times higher in the other treatments compared to the 8/16 h. LUE was higher in all other treatments, with the 12/12 h showing an increase of approximately 2.02 times. Conversely, EUE was approximately 1.32 and 1.02 times lower under the 20/4 and 16/8 h, respectively, compared to the 8/16 h, but 1.35 times higher under the 12/12 h.

### Correlation analysis

**Fig. 7:**
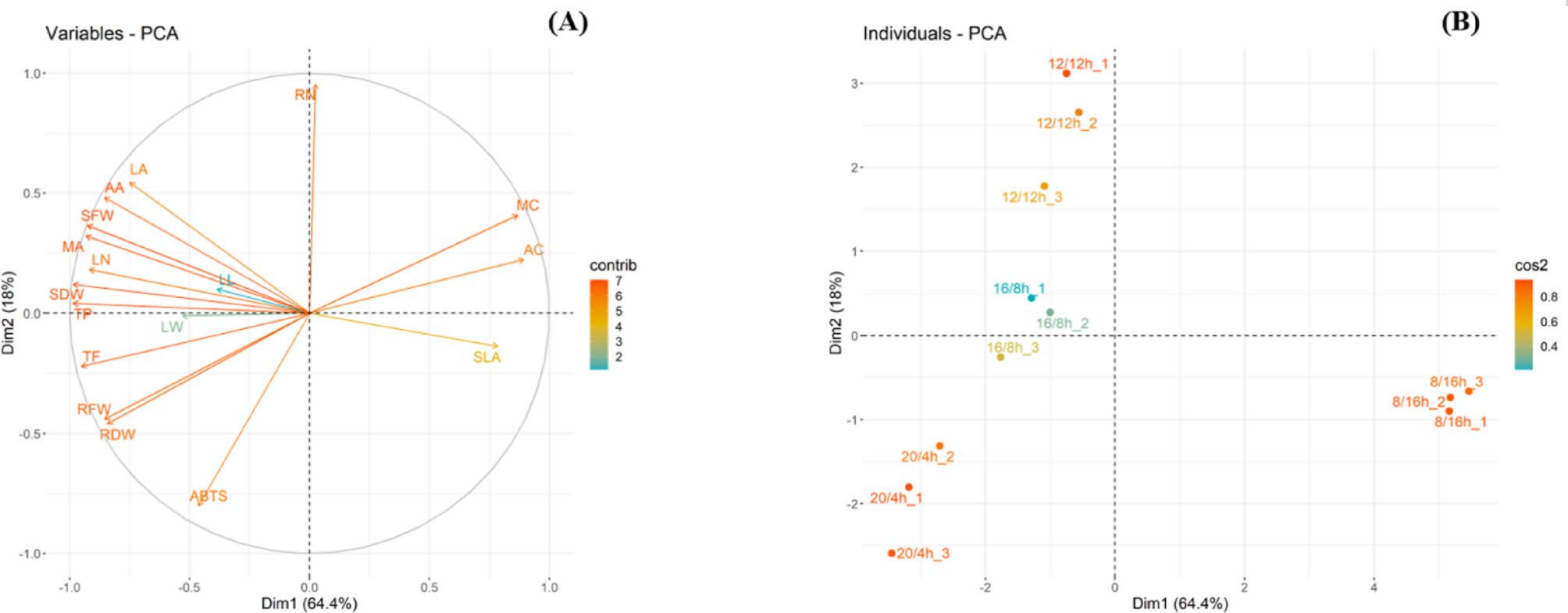
Multivariate principal component analysis (PCA) of the growth characteristics and secondary metabolites of *Centella asiatica* under different photoperiod conditions (20 h light/4 h dark; 20/4 h, 16 h light/8 h dark; 16/8 h, 12 h light/12 h dark; 12/12 h, and 8 h light/16 h dark; 8/16 h). **(A)** Correlation plots summarizing the metabolic relationships among the variables measured under different photoperiods. **(B)** PCA scatter plots showing distinct growth and metabolite patterns in *Centella asiatica* under different photoperiod conditions (*n* = 3). Abbreviations: SFW: shoot fresh weight; RFW: root fresh weight; SDW: shoot dry weight; RDW: root dry weight; LN: number of leaves; SLA: specific leaf area; LA: leaf area; RN: number of runners; LW: leaf width; LL: leaf length; TP: total phenol; TF: total flavonoid; ABTS: ABTS assay; MA: madecassoside; MC: madecassic acid; AA: asiaticoside; AC: asiatic acid. The significance level for the model was set at *p* < 0.05.

To better analyze the impact of photoperiods on the growth of *C. asiatica* and changes in specialized metabolites, PCA was conducted (Fig. 7). PC1 and PC2 together accounted for 82.4% of the total variation, explaining 64.4% and 18%, respectively. In the biplot, the responses of *C. asiatica* under 20/4, 16/8, and 12/12 h photoperiods were distinctly separated from those under the 8/16 h. Notably, the responses of most growth parameters and metabolic substances differed markedly under 20/4, 16/8, and 12/12 h treatments compared to the 8/16 h, except for madecassic acid (MC) and asiatic acid (AC) (Fig. 7A, B).

## Discussion

Photoperiod is a critical environmental factor regulating photosynthetic activity, biomass accumulation, and secondary metabolism in plants^29^. Longer photoperiods generally promote growth, as observed in lettuce cultivars “Rex” and “Rouxai,” which exhibited 62% and 67% higher fresh weights under extended photoperiods^10^. In this study, *C. asiatica* showed similar trends, with fresh weight declining beyond a 12 h photoperiod, whereas dry weight increased linearly with longer photoperiods. Similarly, Liang et al. reported that extending the photoperiod from 16 h to 20 h enhanced biomass accumulation and root vitality in medicinal cannabis^30^, although 24 h of continuous light caused photostress and pigment degradation. Excessive light exposure beyond 12 h, particularly under the 20/4 h treatment, induced physiological disorders such as leaf pigmentation, margin curling, tip necrosis, and chlorosis, likely caused by excessive excitation energy exceeding photosynthetic capacity and leading to photooxidative stress and reactive oxygen species (ROS)^31^. Although photosynthetic parameters were not directly measured, the increase in biomass under photoperiods longer than 12 h suggests enhanced photosynthetic activity, while the visible symptoms at the longest photoperiods (especially 20/4 h) indicate photoinhibition or oxidative stress due to excessive photon exposure. Future studies incorporating chlorophyll fluorescence or gas exchange measurements could clarify the relationship among photoperiod, photosynthetic efficiency, and stress responses in *C. asiatica*.

In this study, fresh and dry root weights were similar to those in a previous studies, in which cuttings rooting of ‘Royal Purple’ increased when the photoperiod was extended from 8 to 16 h^32^. Another study reported that in yellow maca, a 16 h photoperiod compared to 8 h enhanced root fresh and dry weights by approximately 4- and 7-fold, respectively, leading to improved nutrient and water uptake efficiency, which may also influence shoot development^33^. Similarly, extending the photoperiod increased the root biomass of cherry radish by regulating the expression of hormones and genes in response to internal and external signals^34^. Mao et al. reported that a longer photoperiod enhanced root growth, improved LUE, and increased chlorophyll content, resulting in superior growth of lettuce. In addition to photoperiod adjustment, control of light intensity is also necessary for efficient crop production^35^.

As reported in previous studies, plants adjust leaf area and chlorophyll content to optimize light interception and carbon gain under fluctuating light environments^36,37^. In lettuce, leaf number and biomass increased linearly between 12 and 18 h but declined beyond 18 h, and energy efficiency per leaf number decreased sharply after 22 h^38^. These results support that in *C. asiatica*, a photoperiods longer than 12 h may reduce growth efficiency due to stress. Therefore, in this study, a longer photoperiod (light of 12 h or longer compared to 8 h) resulted in a greater number of leaves. Similarly, increasing the photoperiod from 8 to 16 h has been reported to enhance the leaf area of radish^37^. Another study found increased leaf area with photoperiods extended up to 20 h at 150 PPFD^39^. Also, Elkins and van Iersel reported that the leaf area of Rudbeckia seedlings increased with increasing light duration from 12 to 18 h and decreased at 21 h^40^. In this study, the leaf area of *C. asiatica* significantly increased under a photoperiod of 12 h or longer compared to 8 h.

SLA, which represents the surface area capturing light per unit dry mass investment, is an important trait from physiological and biophysical perspectives^41^. When light is abundant, plants invest less energy in leaf mass, resulting in decreased SLA^42,43^. Adams and Langton suggested that increased SLA compensates for a reduced net assimilation rate by enlarging leaf area^44^. Although photosynthetic rate was not directly measured in this study, the high SLA observed under 8/16 and 12/12 h treatments may indicate a morphological strategy for *C. asiatica* to maintain carbon acquisition through leaf area expansion. Nevertheless, high SLA does not necessarily imply high photosynthetic efficiency per unit area; therefore, further measurements of photosynthetic rate and assimilate allocation are required.

Guo et al. reported that runners in strawberries are induced by gibberellin (GA) biosynthetic genes under long photoperiods and low temperatures^45^. In this study, the number of runners was higher at 12/12 h, although no clear pattern was observed based on photoperiod alone. Further research is necessary to investigate runner induction in *C. asiatica*, including combined treatments involving temperature and hormone analyses. These results suggests that *C. asiatica* adapts to limited resources and varying photoperiods by modifying morphological traits, such as leaf characteristics, to optimize growth and survival.

Beyond morphological traits, photoperiod also influenced the accumulation of bioactive compounds. Environmental stimuli such as light intensity and duration regulate the biosynthetic pathways of secondary metabolites by modulating gene expression and enzyme activity^46^. In medicinal plants, the biosynthetic pathways of specialized secondary metabolites are tightly regulated by abiotic signals such as photoperiod, which modulate gene expression and enzyme activities involved in metabolite synthesis. Among these compounds, flavonoids and polyphenols contribute to antioxidant defense and are known for their anticancer and antimicrobial properties^23,47,48^. Previous studies have demonstrated that longer photoperiods in basil enhance light absorption, leading to increased TF and TP accumulation^49^. Similarly, Bian et al. reported that growing lettuce under 24-h light conditions increased phenolic content and free radical scavenging activity, thereby mitigating reactive oxygen species (ROS) caused by carbon accumulation^50^. Photoperiod-induced ROS stress has been reported to trigger an oxidative burst-like response in plants, characterized by increased extracellular peroxidase activity and decreased catalase activity^51^. This indicates that prolonged or altered photoperiods can modulate ROS homeostasis and influence both growth and secondary metabolite accumulation via ROS-mediated signaling pathways. However, conflicting results showed that TP and TF levels in celery cultivars increased up to 12 h of photoperiod but decreased at 16 h, possibly due to photoinhibition or stress caused by prolonged light exposure interfering with chloroplast function and altering metabolic distribution^52^. Additionally, Son and Oh observed an inverse relationship between phenolic accumulation and antioxidant capacity during lettuce growth^26^. In this study, elevated TP, TC, and ABTS values under longer photoperiods may indicate enhanced antioxidant responses to mitigate ROS generated by extended light exposure.

The major specialized secondary metabolites in *C. asiatica* are centelloides, categorized into two groups: Centella saponins (madecassoside and asiaticoside) and sapogenins (madecassic acid and asiatic acid)^53^. These compounds act not only as secondary metabolites mediating plant–environment interactions but also as bioactive substances with pharmacological activities such as wound healing, anti-inflammatory, and neuroprotective effects^54,55^. In this study, it was shown that photoperiod significantly affected centellosides content. Previous research indicated that high light growth conditions at 1270 µmol·m⁻²·s⁻¹ induce oxidative stress in *C. asiatica* compared to low light conditions of 300 µmol·m⁻²·s⁻¹, which promotes the biosynthesis of saponins such as madecassoside and asiaticoside^22^. The biosynthesis of these saponins is regulated by glycosylation of sapogenins, affecting their stability, storage, solubility, biological activity, and intracellular transport signaling^56^. Although photosynthetic or oxidative stress indices were not measured, madecassoside and asiaticoside contents peaked at 16/8 h, while madecassic acid and asiatic acid were highest at 12/12 h, suggesting that photoperiod-induced physiological responses promoted metabolite accumulation (Table S1). This study focused on photoperiod effects on *C. asiatica*, while the impact of light spectrum, especially red-to-blue ratios, is further examined in the following experiments. The results indicated that red light with 20% blue light yielded the highest centellosides content, while higher proportions of blue light promoted increases in total phenolic content, total flavonoid content, and antioxidant activity^23^. These results demonstrate that the production of bioactive compounds in *C. asiatica* can be optimized through specific photoperiods. For more comprehensive optimization, future studies should also consider additional environmental factors such as light spectrum, light intensity, and nutrient availability.

Effective use of artificial lighting is a critical factor for ensuring economic feasibility and environmental sustainability in vertical farms^28^. Nguyen et al.^57^ reported growers increasingly focus on LUE, which is closely associated with LED lighting performance and plant growth. In this study, shoot fresh weight increased with longer photoperiods, although this did not correspond to higher energy utilization efficiency. Using 8/16 h as the baseline, which had the lowest biomass content, the 12/12 h achieved the highest LUE and EUE at the same PPFD. Unlike previous studies that calculated LUE and EUE based on fresh weight, this study used dry weight to better represent actual biomass accumulation and physiological efficiency. Previous studies also reported that when photoperiods were set at 16, 20, and 24 h under the same PPFD, crops like lettuce and chicory exhibited higher LUE due to increased biomass characteristics, such as leaf area at 16 h. Conversely, basil showed no improvement in biomass, and EUE decreased as the photoperiod extended^28^. Similarly, in this study, shoot biomass of *C. asiatica* increased with photoperiod duration, and LUE increased at 12 h or longer, while EUE increased only at 12 h. These results suggest that a 12 h photoperiod provides an optimal balance between biomass accumulation and energy efficiency for cultivating *C. asiatica* under 200 µmol·m⁻²·s⁻¹ white LED lighting.

Photoperiod strongly influences plant responses, and its effects can be further understood through the total light dose, represented by the daily light integral (DLI). Variations in photoperiod directly affect DLI, which quantifies the total photosynthetically active radiation received per day based on light intensity and duration^58^. Previous studies on DLI have reported that in *M. crystallinum*, morphological traits such as dry weight and leaf area increased with higer DLI, whereas excessive DLI induced physiological stress by reducing chlorophyll content^59^. In another study, Xu et al. observed that when DLI increased from 17.3 to 34.6 mol·m⁻²·d⁻¹ under continuous light (24 h), biomass and leaf area increased in Nasturtium, suggesting morphological adaptation to optimize photosynthetic resource use^60^. The biomass and morphological differences observed in this study are consistent with these adaptive responses. For instance, at a constant PPFD of 200 µmol·m⁻²·s⁻¹, increasing the photoperiod from 8 h to 12 h proportionally increased DLI from 5.76 to 8.64 mol·m⁻²·d⁻¹ (16 h: 11.52 mol·m⁻²·d⁻¹, 20 h: 14.4 mol·m⁻²·d⁻¹). Although photosynthetic rate was not directly measured, the DLI-dependent changes in leaf characterics likely ehanced photosynthetic activity and influenced metabolite partitioning.

In this study, when the photoperiod exceeded 12 h (DLI: 11.52–14.4 mol·m⁻²·d⁻¹) at a light intensity of 200 µmol·m⁻²·s⁻¹, physiological disorders such as leaf pigmentation, leaf margin curling, leaf tip necrosis, and intermittent chlorosis occurred, resulting in quality deterioration despite increased shoot growth. This suggests that exceeding a certain DLI threshold does not proportionally enhance growth but rather induces light stress, potentially impairing energy utilization efficiency and quality. These physiological disorders likely resulted from inefficient use of absorbed light energy and the functional burden on chloroplasts caused by ROS-induced oxidative stress. Although oxidative stress was not directly measured, but was indirectly evaluated through antioxidant indicators such as TP, TF, and ABTS. These results suggest that *C. asiatica* may be highly sensitive to photoperiod and DLI, highlighting the need for an integrated approach encompassing photoreceptor activity, oxidative stress–related metabolites, and precise photoperiod control.

Changes in the photoperiod inherently alter the duration of the dark period. During the dark period, plants maintain growth and regulate carbon balance primarily through respiration, which depends on the activity of pathways such as the tricarboxylic acid (TCA) cycle and the mitochondrial electron transport chain^61^. These pathways involve key enzymes such as pyruvate kinase and succinate dehydrogenase, and recent evidence suggests that their activity is also regulated by the circadian rhythm^62^. Therefore, future studies should integrate analyses of photoperiod conditions, dark period duration, and circadian rhythm regulation to develop a more precise light environment–based production strategy for *C. asiatica*.

## Conclusion

This study demonstrated the effects of photoperiod variations on the growth and centellosides biosynthesis of *C. asiatica* under controlled environments. A 12/12 h photoperiod provided the best balances among biomass accumulation, centellosides production, and energy-use efficiency, whereas extended light exposure (> 12 h) induced physiological stress symptoms. These findings highlight the importance of precise photoperiod regulation to maximize productivity and maintain plant health. Future studies should explore the combined effects of photoperiod and other environmental parameters, such as light spectra and nutrient availability, to further optimize *C. asiatica* cultivation for pharmaceutical and cosmeceutical applications.

## Supplementary Information

Below is the link to the electronic supplementary material.

Supplementary Material 1

## Data Availability

The datasets generated during and/or analyzed during the current study are available from the corresponding author upon reasonable request.
