# Supplementary material for: Optimizing photoperiod for growth and centellosides biosynthesis in Centella asiatica under vertical farming conditions
Source: Sci Rep. 2026 Mar 22;16:14537. doi: 10.1038/s41598-026-44883-w (PMC13153366; doi:10.1038/s41598-026-44883-w)
Supplement: Supplementary file 1 — Supplementary Material 1 [file 41598_2026_44883_MOESM1_ESM.docx]

**Supplementary Table S1.** Centelloside content measurement results of 4 treatments of *Centella asiatica..*

| **Photoperiod**  **(day/night)** | **Madecassoside** | **Asiaticoside** | **Madecassic acid** | **Asiatic acid** |
| --- | --- | --- | --- | --- |
| 20/4 h | 12.94 ± 0.01^z^ b^y^ | 19.69 ± 0.01 c | 0.71 ± 0.01 d | 0.97 ± 0.00 c |
| 16/8 h | 13.04 ± 0.02 a | 21.04 ± 0.07 a | 1.06 ± 0.02 c | 1.34 ± 0.00 c |
| 12/12 h | 12.34 ± 0.01 c | 20.81 ± 0.00 b | 2.00 ± 0.03 a | 2.38 ± 0.12 a |
| 8/16 h | 3.18 ± 0.00 d | 5.46 ± 0.00 d | 1.24 ± 0.02 b | 1.84 ± 0.50 b |

^z^Means ± S.D.; ^y^Means with different letters within a column are significantly different (*p* < 0.05)

| **Photoperiod**  **(day/night)** | **LED power**  (W) | **Daily energy consumption**  (kWh day^−1^) | **Daily electricity cost**^z^  (USD/day) | **4-Week electricity cost**^z^  (USD/28 days) |
| --- | --- | --- | --- | --- |
| 20/4 h | 25 | 0.50 | 0.107 | 3.00 |
| 16/8 h | 25 | 0.40 | 0.086 | 2.40 |
| 12/12 h | 25 | 0.30 | 0.064 | 1.80 |
| 8/16 h | 25 | 0.20 | 0.043 | 1.20 |

**Supplementary Table S2.** Daily and 4-week electricity consumption and costs for single LED fixtures at various photoperiods.

^z^Daily electricity costs were estimated based on the average electricity rate in Korea (~₩150/kWh) and are presented in USD for international reference (~0.11 USD/kWh).


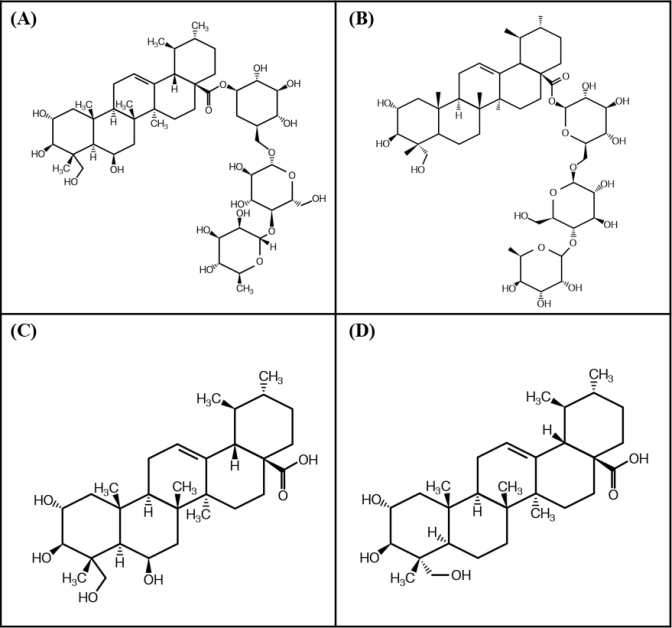


**Supplemental Figure S1.** Chemical structures of the major bioactive compounds identified in *Centella asiatica* (A, madecassoside; B, asiaticoside; C, madecassic acid; D, asiatic acid).


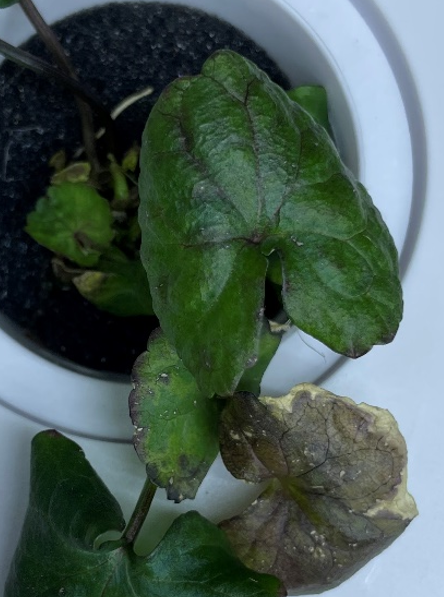


**Supplemental Figure S2.** Physiological damage on *Centella Asiatica* leaves, showing discoloration and necrosis.
